# Indiscriminate slaughter of pregnant goats for meat in Enugu, Nigeria: Causes, prevalence, implications and ways-out

**DOI:** 10.1371/journal.pone.0280524

**Published:** 2023-01-17

**Authors:** Patience C. Ugwu, Emmanuel O. Njoga, Ugochinyere J. Njoga, Chinwe J. Aronu, Everest O. Atadiose, Chinwe E. Okoli, Onyinye S. Onwumere-Idolor, Festus E. Ajibo, Nichodemus N. Azor, Sunday N. Bernard, Ikenna E. Ozioko, Ikechukwu S. Eze, Festus O. Abonyi

**Affiliations:** 1 Department of Animal Health and Production, Faculty of Veterinary Medicine, University of Nigeria, Nsukka, Nigeria; 2 Department of Veterinary Public Health and Preventive Medicine, Faculty of Veterinary Medicine, University of Nigeria, Nsukka, Nigeria; 3 Department of Veterinary Obstetrics and Reproductive Diseases, Faculty of Veterinary Medicine, University of Nigeria, Nsukka, Nigeria; 4 Department of Veterinary Public Health and Preventive Medicine, Faculty of Veterinary Medicine, University of Abuja, Federal Capital Territory, Abuja, Nigeria; 5 Department of Animal Production, Faculty of Agriculture, Delta State University of Science and Technology, Ozoro, Delta State, Nigeria; 6 Department of Animal Health and Production, Enugu State Polytechnic, Enugu, Nigeria; North Carolina State University, UNITED STATES

## Abstract

**Background:**

The indiscriminate slaughter of pregnant goats (SPGs) undermines meat production and food security especially in developing countries. It also connotes animal cruelty, depletion of goat population and may enhance the spread of zoonotic pathogens inhabiting the female reproductive tract during carcass processing. Consequently, this study determined the causes and prevalence of slaughtering pregnant goats for meat in Enugu, Nigeria. The study also estimated the economic losses associated with SPGs, discussed the negative public health consequences and suggested the ways-out.

**Methods:**

Structured, validated and pilot-tested questionnaire was used to ascertain the reasons for SPGs for meat among 78 willing and randomly selected respondents. The questionnaire survey was conducted in the form of interview. Pregnancy statuses of the goats slaughtered were ascertained by visual inspection and palpation of the eviscerated and longitudinally incised uteri and the horns for macroscopic evidence of pregnancy. Ages of the dams were estimated by dentition method. Estimation of the gestational age was performed by crown-rump length method. The study lasted for six months, comprised of three months (December to March) during the dry/hot season and another three months (May to August) during the wet/rainy season. Economic loss estimation was based on the current monetary values of a matured (30 kilogram) goat and one kilogram of chevon in Enugu, Nigeria; which was determined through market survey. Pearson’s Chi-square test was used to determine whether there were significant (P<0.05) statistical associations between SPGs and age and season.

**Results:**

Major reasons adduced for SPGs were: economic hardship (41%), ignorance of the goat’s pregnancy status (21%), increased demand for chevon (13%) and feed scarcity during drought (11%). Of the 1,658 does examined during the six months study, 589 (35.5%) were pregnant. The majority (876/1658, 52.8%) of the female goats slaughtered were in their active reproductive age of ≤ 4 years, while 782 (47.2%) were aged > 4 years. Similarly, majority (1007/1658, 60.7%) of the does/nannies were slaughtered during the dry/hot season. A total of 907 foetuses at first (n = 332, 36.6%), second (n = 486, 53.6%) and third (n = 89, 9.8%) trimesters of gestation were recovered from the 589 PGs. Singleton, twin and triplet pregnancies were observed in 312 (53%), 236 (40%) and 41 (7%) PGs, respectively. About ₦34.44 million ($83,390) would have been earned if the foetuses were born alive and raised to maturity. Additionally, 19,136 kg of chevon, valued at ₦47,841, 000 ($115,838), which would have accrued from the wasted foetuses was also lost.

**Conclusion:**

Considering the economic, zoonotic and livestock production implications of this work, frantic efforts to reduce SPGs in Enugu, Nigeria is imperative. This could be achieved through advocacy, goat farmers’ enlightenment, ante-mortem pregnancy diagnosis, provision of subsidized feed materials during the dry season and strict enforcement of the Nigerian Meat Edict law, which proscribes unapproved slaughter of gravid animals. These measures may improve food safety and security, improve goat reproduction and production, reduce protein malnutrition, limit dissemination of zoonotic pathogens during carcass processing and hence protect public health in Nigeria.

## Introduction

The domestic goat, *Capra aegagrus hircus*, belongs to the genus *Capra* and the family *Bovidae*. In most agrarian African settings, ownership of livestock, especially goats, is a measure of social status, cultural heritage, and form of insurance against crop failure [1]. Consequently, various goat breeds—Sahel, Maradi, Red Sokoto, Kano brown and West African Dwarf (WAD)—are widely reared for production of meat, milk or skin in Africa [2–5]. In Nigeria, people often refer to the goat as the “cattle of the poor” because of its importance in the provision of animal protein and the farmers’ economic wellbeing. Most African indigenous goat breeds are prolific, can survive on available shrubs and trees, and thrive in adverse tropical environment where crop farming is impossible [6, 7]. Goat production is less capital intensive and enjoys greater economy of space. Therefore, subsistent or medium-scale goat farming is practiced in almost all rural households in Enugu State, Nigeria; as people rely on the proceeds to offset family expenses or to augment income. Moreover, goat and the meat products are readily marketable in Enugu State, Nigeria as chevon is relished, even among elites, as a special native delicacy called “isi-ewu” in the Eastern part of Nigeria [8].

Despite the importance of goats and its comparative advantages over cattle, goat farming is not thriving as expected in Nigeria as there are 99.9 million goats in the country [9]; notwithstanding the widespread goat rearing activities in most households. The causes of the unthriftiness in goat production may be multifactorial, but the slaughter of pregnant goats (SPGs) for meat is undoubtedly one of the major causes. Rampant SPGs threatens food (meat) security and connotes animal cruelty [1, 10]. Enormous economic, foetal and livestock-resources wastages inherent in the slaughter of pregnant female animals (SPFAs) for meat [1], make SPGs counterproductive in many ways. Indiscriminate SPGs for meat not only depopulate reproductive females (through excessive off-takes) but also represents the future herd extermination via the consequential and unwarranted foetal wastages. There is also the demerit of irretrievable loss of good genetics in the wasted foetuses, which would have been used to crossbreed and enhance productivity in other goat breeds like the WAD. Therefore, SPGs jeopardizes efforts toward achieving food security and self-sufficiency in provision of animal proteins; particularly in Nigeria with a huge human population estimated at 217 million (as at August 2022) and experiencing one of the fastest national population growths in the world [11].

Apart from diminution in available edible animal protein, SPGs may facilitate the introduction of exotic zoonoses through meat importation due to the reduction in the amount of meat and other locally produced foods of animal origin [1]. The SPGs may also aggravate spread of zoonoses, especially in the tropics, where favourable climatic conditions for survival and proliferation of pathogens abound [12, 13]. Transmission of *Toxoplasma*, *Brucella*, *Listeria*, and zoonotic *Staphylococcus* species is possible via wound contamination during foetal evisceration or processing of the reproductive tract of infected animals [14–18]. Moreover, the processing of pregnant goats (PGs) carcasses infected with zoonotic pathogens may result in contamination of the meat or the abattoir environment for onward transmission to humans or other susceptible animals in the vicinity [19]. This may facilitate and even perpetuate the zoonotic transmissions/infections among humans, animals and the environment.

As a result of the need to conserve livestock resources and improve animal welfare, the Nigerian Meat Edict of 1988 prohibits the SPFAs, except for emergency slaughter, to relieve animal suffering, as may be approved by Veterinarians. However, the law is both obsolete and poorly implemented such that the SPFAs has continued unabated. Recently published reports [20–23] show that the SPGs for meat has persisted in some parts of the country over the years. Given the plethora of problems associated with indiscriminate slaughter of reproductive female animals for meat, it is worthwhile to ascertain the current status of this practice and unravel the drivers underpinning it in Enugu, Nigeria; where most rural households engage in small scale goat farming and depend of the proceeds to augment their family income. Therefore, this study determined the causes and prevalence of slaughtering PGs for meat in Enugu, Nigeria, in order to discuss the negative consequences (foetal wastages, food insecurity, economic losses and possible spread of zoonoses) and make evidence-based recommendations to solve these problems. This has become imperative considering that there is no published report on SPGs in Enugu metropolis, Nigeria to the best of our knowledge. Additionally, the number of gravid goats slaughtered in the study area may give useful information on compliance to the Meat Edict, a law that forbids SPFAs in Nigeria. The root causes of SPGs, if determined, will guide policy formulation to forestall maternal slaughter for meat in order to boost goat production, conserve livestock resources, limit zoonoses spread and ensure food security and safety.

## Materials and methods

### Ethical approval and statement of informed consent

Ethical approval (Ref No: VPHPM/UNN/22/21) to carry out this work was granted by the Research Ethics Committee of the Department of Veterinary Public Health and Preventive Medicine, University of Nigeria, Nsukka; on August 26, 2021. Oral informed consent to partake in the questionnaire survey was requested and obtained from all the participants surveyed. At first, the leadership of Enugu goat slaughters’ associations verbally approved that we interview/survey their members during our familiarization and consultative meetings with them. Additionally, we sought and obtained individual consent to partake in the study from all the 78 respondents surveyed, in line with the terms and conditions of the ethical approval granted. All the respondents confessed that they were aged ≥ 18 years.

### Study area and study design

The study was carried out in Enugu, Southeast; Nigeria. Enugu metropolis is located on latitude 6° 27’ 10"N and longitude 7° 30’ 40"E [24]. The majority of the population are civil servants, traders or agrarian farmers but food-animal production, as an additional source of income, is widely practiced. A cross-sectional study design, involving questionnaire survey to determine the causes of SPGs for meat and determination of the pregnancy statuses of does (female goats) slaughtered in the study area, was carried out. Additionally, economic and meat/chevon losses due to the resultant foetal wastages were estimated based on the current monetary values of an average matured goat (30 kilogram live weight) and a kilogram of chevon determined via market survey.

### Research visit and sample size determination

Two major goat slaughterhouses in Enugu metropolis (Akwata and Artisan), where goat carcasses are mostly processed were purposely selected and visited every other week for data collection. The study lasted for six months, which consisted of three months (December 05, 2021 to March 04, 2022) during the dry/hot season and another three months (May 02, 2022 to August 01, 2022) during the wet/rainy season. A minimum sample size of 385 goats was calculated for the study using Raosoft^®^ sample size calculator available at: http://www.raosoft.com/samplesize.htm [25]. The sample size computation assumed an estimated target population size of 20,000 goats (since there is no record of the current population of goats in Enugu, Nigeria; to the best of our knowledge), 5% error margin, 95% confidence level and 50% prevalence. Nevertheless, a total of 1,658 does selected (from 2,560 slaughtered female goats) by simple random sampling technique (SRST), through the toss of a coin, were examined for accuracy and robustness of data.

### Questionnaire survey

#### Validation

At first, the structured questionnaire was subjected to face and content validations as described by Bolarinwa [26]. Afterwards, a three-man panel of veterinary experts reviewed the questionnaire. The experts also assessed and scored each question on the basis of relevance and clarity, using a 4-point scale, and made recommendations accordingly. From the scores, the scale-cumulative validity index (s-CVI) and the mean item-cumulative validity index (mean i-CVI) were computed according to the method previously described by Zamanzadeh et al. [27]. Questions that its s-CVI or i-CVI values were less than 0.9 were revised as recommended to enhance both relevance and clarity. Thereafter, the scores were recomputed and both the s-CVI values for relevance and clarity were unity. Similarly, the mean i-CVI for relevance and clarity were 0.97 and 1 respectively. A copy of the questionnaire validity assessment scores is available as supplementary file. Moreover, Cronbach’s Alpha test was carried out to further ascertain the questionnaire’s validity in obtaining the parameters of interest. The test yielded an alpha-value of 0.88 (88%), which was greater than the 0.7 (70%) benchmark. Afterwards, the reliability/consistency of the questionnaire was ascertained by the test-retest method [28]. Finally, the pilot testing of the questionnaire was done using 20 respondents (from Nsukka slaughterhouse, Nigeria) prior to the real survey, and inconsistencies observed were corrected. Responses from the pilot test were not included in the actual survey.

The questionnaire sought to elicit information on: (1) the socio-demographics (age, gender, education status, job description, working experience and training on modern goat production or processing practices) of respondents; (2) possible causes of SPGs for meat and (3) major method of disposal of eviscerated foetuses or the gravid uteri contents. A copy of the questionnaire is available as a supplementary material. From a sampling frame of 102 (determined by head count of those who reported for work on the day of the survey in the two slaughterhouses), 78 respondents (butchers/goat meat sellers and goat farmers/sellers) who willingly volunteered to partake in the study, were all surveyed. At the two selected slaughterhouses, the questionnaire survey was done just once but on different days. Before the survey, the respondents were assured of the confidentiality of the responses. The survey was conducted in the form of an interview schedule, in full compliance with the Helsinki declaration of the World Medical Association 2013 [29]. During the survey, extreme care was taken to ensure that each respondent was surveyed just once.

#### Pregnancy statuses of the slaughtered does

Pregnancy statuses of the goats slaughtered were ascertained by Veterinarians through visual inspection and palpation of the eviscerated and longitudinally incised uteri and the horns for macroscopic evidence of pregnancy–foetuses, placentas, placentomes, etc. During the procedure, personal protective equipment, including coverall, hand gloves, nose and mouth mask, eyes goggle–were used to prevent possible zoonotic infections. Ages of the dams were estimated by dentition method. Estimation of the gestational age of the foetuses was performed by crown-rump length measurement using the formula: X = 2.1 (Y + 17); where X = Age of foetus in days and Y = Crown-rump length in cm as described by Arthur et al. [30]. Since the average gestational period of goats is 150 days, the gestational age was then classified as first (≤ 50 days), second (51–100) and third (>100 days) trimesters, respectively. Similarly, sexes of the foetuses were determined by visual examination of the external genitalia at the inguinal region and below the base of the tail.

#### Estimation of losses due to slaughter of pregnant goats

Economic wastages associated with foetal wastage were estimated in monetary terms as revenues that would have accrued from the sale of the goats on maturity [31]. The amount of chevon that would have accrued from the wasted foetuses was estimated based on 74% carcass yield (inclusive of offals which are edible and relished in Nigeria) and an average maturity live weight of 30 kilogram per goat as described by Casey et al. [32]. The estimation assumed that the foetuses would be born alive and raised to maturity but allowance for 5% pre-maturity mortality and costs of raising the foetuses to maturity were factored-in, as recommended by Ndi et al. [31] and Casey et al. [32]. The monetary value was estimated in Nigerian currency, Naira (₦) and converted to US Dollar ($) based on the official (Central bank of Nigeria) exchange rate of ₦413 per US Dollar as at January 2022 [33]. Gross revenue losses associated with SPGs were determined based on the minimum market prices of ₦40,000 ($96.9) and ₦2,500 ($6.1) for an average matured goat and one kilogram of chevon respectively in the study area as at January 2022. Since goats are organically reared in most parts of Africa and to compensate for any possible costs of rearing the kids to market weight, the least (minimum) market prices of a mature goat and the cheapest unit price of chevon per kilogram (determined during the market survey) were used in the economic loss estimation as recommended [31].

#### Data analysis and presentation

Information obtained from the questionnaire study were collated and presented in tables and charts. Pearson’s Chi-square test was used to ascertain whether there is an association (p<0.05) between SPGs and season and age of the dams. The statistical analysis was performed at 5% probability level using GraphPad prism^®^, version 8.4.3 (GraphPad Inc., San Diego, California, USA).

## Results

### Socio-demographics of goat sellers and the carcass processors

The 78 respondents, which consisted of 32 goat farmers/sellers and 46 butchers/carcass processors/meat sellers, were all males and claimed to have had training on modern livestock production/slaughterhouse practices. The majority (64.1%) of the respondents had 6–10 years working experience while 30.8% and 5.1% had < 5 years and > 10 years work experience respectively. On educational status, 38.5% of the respondents had no formal education while 35.1% and 25.6% attained primary and secondary educational levels, respectively.

### Reasons for slaughter of pregnant goats for meat

**Fig 1:**
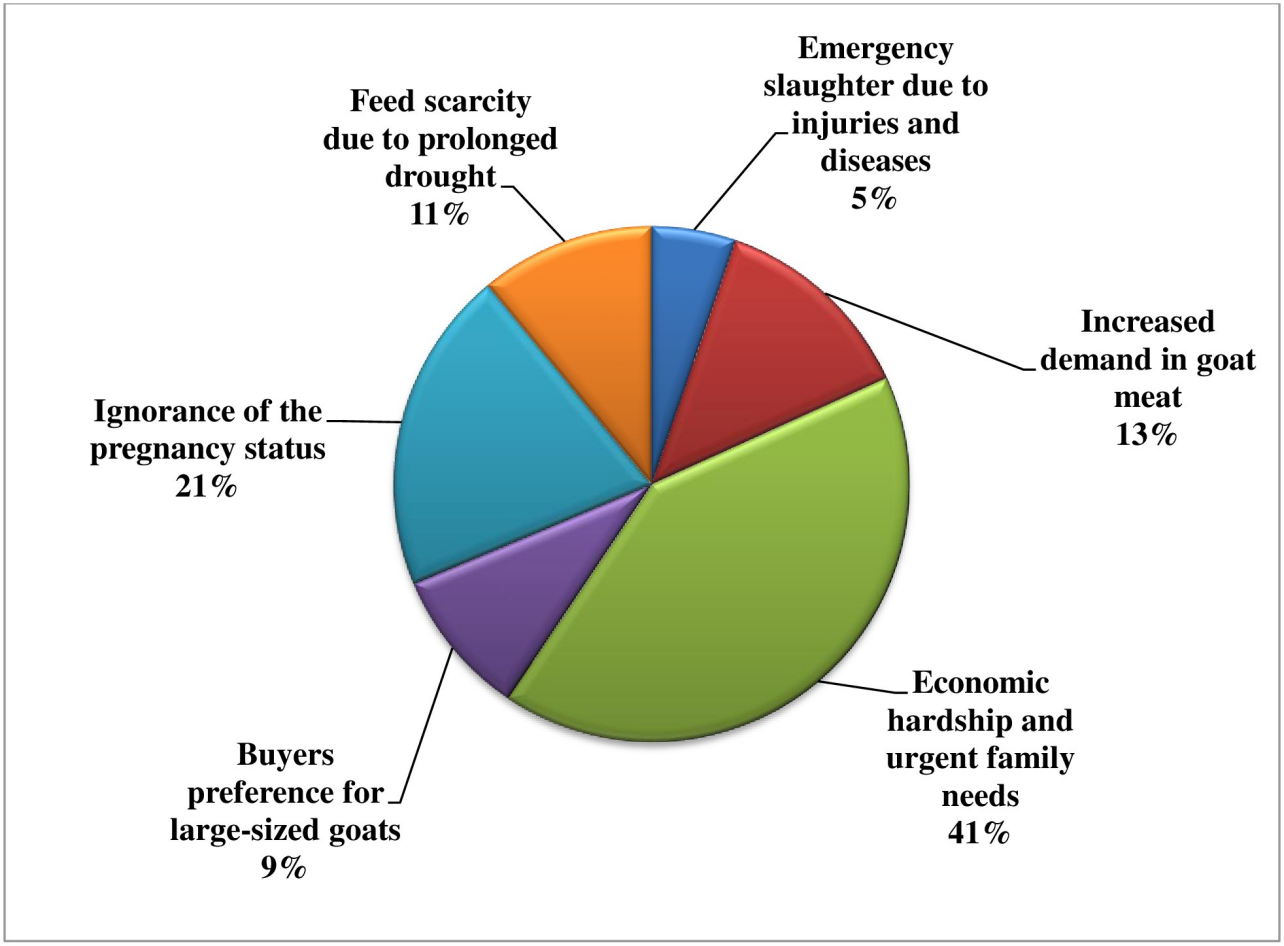
Reasons advanced for sale or slaughter of pregnant goats for meat in Enugu, Nigeria.

Major reasons given for sale/slaughter of PGs for meat were economic hardship (41%), ignorance of their pregnancy status (21%), increased demand for chevon (13%) and feed scarcity during prolonged droughts (5%) as shown in Fig 1.

### Age, breed, seasonal distribution, and pregnancy status of does slaughtered

**Fig 2:**
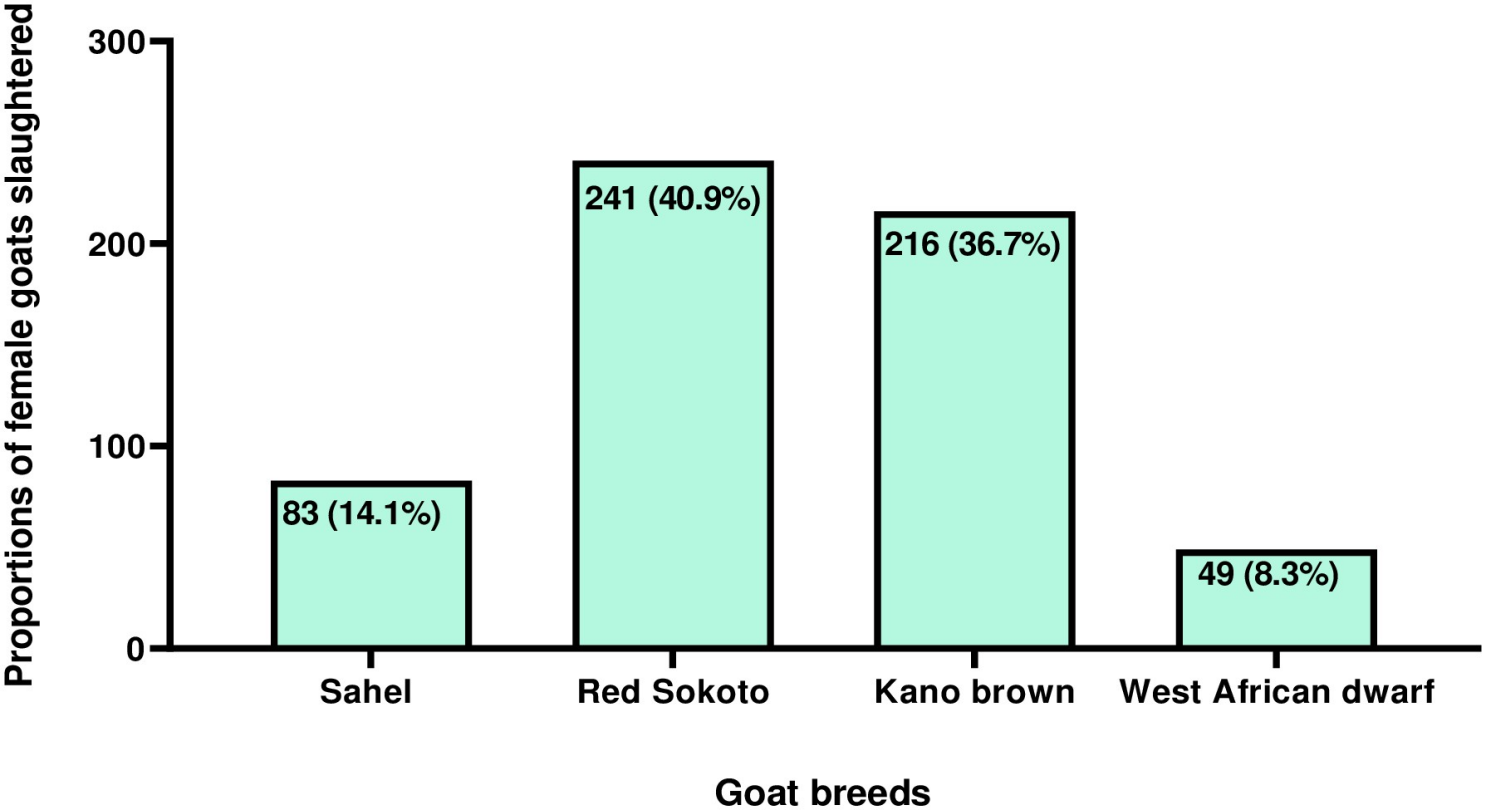
Breed distribution of pregnant goats (n = 589) slaughtered in Enugu, Nigeria.

**Fig 3:**
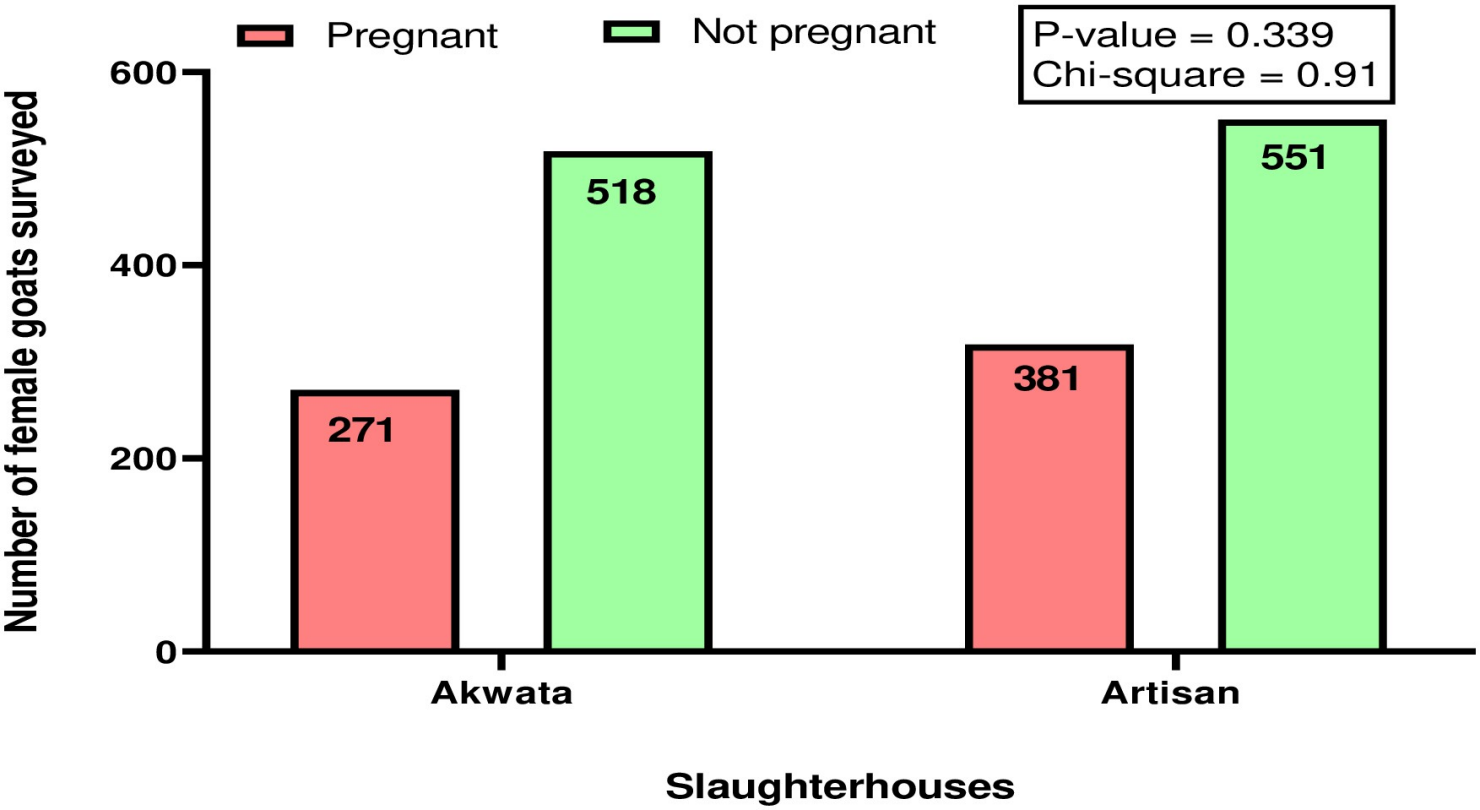
Pregnancy statuses of female goats (n = 1658) slaughtered for meat at Akwata (n = 789) and Artisan (n = 869) slaughterhouses in Enugu, Nigeria.

Of the 2,560 female goats slaughtered during the six months survey, 1,658 were selected and surveyed. The majority (52.8%, 876/1,658) of the selected female goats slaughtered were in their active reproductive age of ≤ 4 years while the rest were aged > 4 years old (Table 1). The result on the breed distribution of the pregnant goats slaughtered is presented in Fig 2. Similarly, the majority (60.7%, 1007/1658) of the nannies were slaughtered during the dry/hot season (December to March) while the rest were killed during the wet/rainy season (May to August). Further details on the seasonal distribution and pregnancy status of female goats slaughtered in the study area are presented in Table 2. There was statistically significant association (P<0.05) between SPGs and age but no significant association was found (P>0.05) between SPGs and season. Of the 1,658 female goats surveyed, 35.5% (589) were pregnant. The proportions of PGs slaughtered in the two slaughterhouses are presented in Fig 3. No statistically significant association (p>0.05) existed between SPGs and the slaughterhouses.

**Table 1:** Age distributions and pregnancy status of female goats slaughtered for meat in Enugu, Nigeria.

Age	Number (%) of does surveyed	Number pregnant	Prevalence (%)	Chi-square	P-value
≤ 4 years	876 (52.8)	288	32.9	5.69	0.02*
> 4 years	782 (47.2)	301	38.5		
Total	1,658 (100)	589	35.5		

*Statistically significant P-value; Pearson’s Chi-square test, GraphPad Prism^®^, version 8.4.3 (GraphPad Inc., San Diego, California, USA)

**Table 2:** Seasonal distribution and pregnancy status of female goats slaughtered for meat in Enugu, Nigeria.

Season	Number (%) of does slaughtered	Number pregnant	Prevalence (%)	Chi-square	P-value
Rainy/wet season	651(39.2)	271	41.6	2.38	0.12
Dry/hot season	1007 (60.8)	318	31.6		
Total	1658 (100)	589	35.5		

Peason’s Chi-square test, GraphPad Prism^®^, version 8.4.3 (GraphPad Inc., San Diego, California, USA)

### Characteristics of caprine foetuses found

A total of 907 foetuses were recovered from the 589 pregnant goats surveyed. Single, twin and triplet pregnancies were observed in 312, 236 and 41 of the PGs respectively. Most (486) of the recovered foetuses were in their second trimester of gestation (51–100 days). Further details on the distributions of the pregnant goats (n = 589) slaughtered and the foetuses (n = 907) recovered according to their gestational age are shown in Table 3. Analysis of the sexes of the foetuses showed that 56% (508/907) were females while 44% (399/907) were males.

**Table 3:** Gestational age distributions of pregnant goats slaughtered and the corresponding number of foetuses recovered at Akwata and Artisan slaughterhouses in Enugu, Nigeria.

Gestational age	Number of dams surveyed (%)	Number of foetus found (%)	Number of multiple pregnancy*
First trimester (1–50 days)	226 (38.4)	332 (36.6)	102
Second trimester (51–100 days)	301(51.1)	486 (53.6)	159
Third trimester (≥101 days)	62 (10.5)	89 (9.8)	16
Total	589 (100)	907 (100)	277

*Multiple pregnancy = Twins (236) and triplets (41)

### Method of foetus disposal

**Fig 4:**
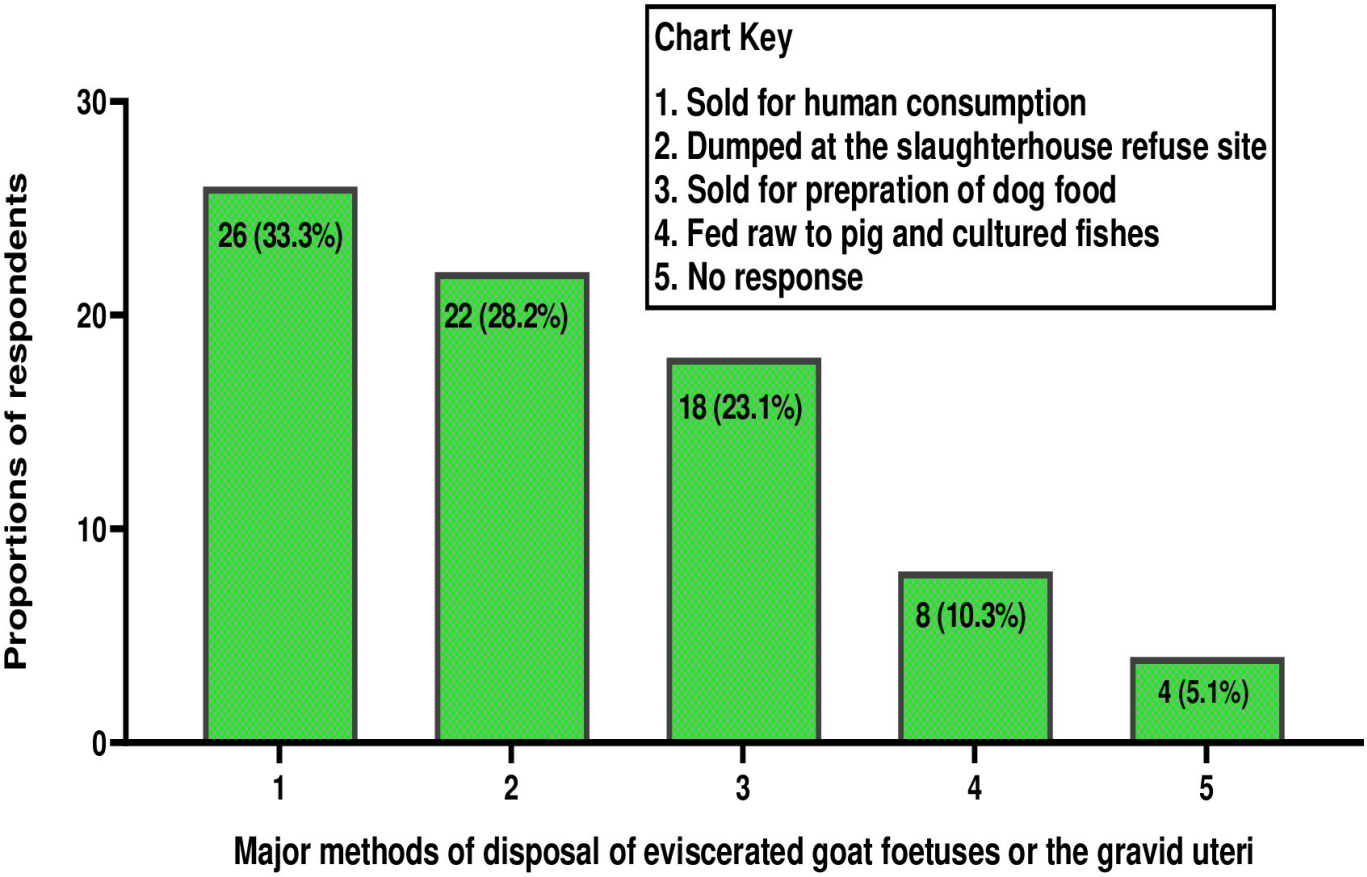
Major methods of disposal of caprine foetuses or gravid uterine content among slaughterhouse workers (n = 78) in Enugu, Nigeria.

Twenty eight percent (22/78) of the slaughterhouse workers disposed the eviscerated foetuses in municipal refuse collection bins/sites. Twenty-three percent (18/78) and 33.3% (26/78) sold the foetuses for preparation of dog food and as meat for human consumption respectively. Other results on methods of disposal of caprine foetuses or the gravid uterine content are presented in Fig 4.

### Economic losses

During the market survey, the price of a 25–35 kilogram female goat and one kilogram of chevon ranged from ₦40,000 ($96.9) to ₦52,000 ($125.9) and ₦2,500 ($6.1) to ₦3,000 ($7.3) respectively in Enugu, Nigeria as at January 2022. An estimated gross income of ₦34.44 million ($83,700), based on a conservative market price of ₦40,000 ($96.9) per average matured goat in the study area, was lost to SPGs during the six months study. Additionally, 19,136 kilogram of chevon, valued at ₦47,841, 000 ($115,838), which would have accrued from the wasted foetuses was also lost. This will amount to an annual loss of ₦95, 682, 000 ($231,675) to caprine foetal wastages in the study area.

## Discussion

As found in this study, the slaughter of 35.5% of PGs is unacceptably high from animal production and food security perspectives, especially in Nigeria where the demand for AP lags the supply [1]. This translates to the wastage of at least one foetus or more (depending on singleton or multiple pregnancies) for every three PGs slaughtered. The SPGs not only depopulates reproductive females, which are the bedrocks in livestock production; but it also indicates the elimination of the future national goat population through the consequential and unwarranted foetal wastages. When active reproductive female goats are slaughtered, up to 52.8% as found in this study, and there are no assisted reproductive technologies in place to aid reproduction in surviving does, the growth of the national herd size may be significantly reduced [34–36]. The statistically significant association found between age and pregnancy status suggests that the greater the number of young goats (≤ 4 years) slaughtered, the higher the chances of SPGs and therefore the more the number of foetuses wasted. Excessive off-takes from the national goat herd, inherent in the SPGs and the resultant foetal losses, without commensurate replacement may lead to animal-protein-food insecurity. This could result in an acute shortage of animal protein and worsen the country’s already precarious food security situation, especially in rural areas where protein malnutrition is glaring [37]. Considering that most of the PGs slaughtered (Sahel, Red Sokoto and Kano brown) were better meat and milk breed than the WAD (which is more tolerant to *Trypanosoma* and *Haemonchus contortus* species infections [38, 39], the foetal wastages imply irretrievable losses of good genetics in the foetuses that could have been used for cross-breeding the WAD for enhanced meat and/or milk production.

Except on the grounds of health or animal welfare considerations, as may be approved by Veterinarian, the SPFAs is unethical [40] and prohibited in Nigeria under the Meat Edict of 1988. Regrettably, indiscriminate sale and slaughter of gravid animals has persisted in Nigeria, despite the negative implications [1, 10]. The SPGs during the second and third trimesters of gestation is a serious concern as pregnancies at these stages can easily be detected, even by visual assessment. Nevertheless, this could be due to economic hardship or fodder scarcity during the dry/hot season. Drought related feed or water scarcity, usually compels livestock farmers to salvage their animals by selling them off, irrespectively of their pregnancy statuses [10]. Additionally, poor ante-mortem inspection of slaughter-animals in some Nigerian slaughterhouses, due to inadequate number of trained personnel may have contributed to the high number of PGs slaughtered and foetuses wasted. Furthermore, poor implementation of the Meat Edict, which proscribes the slaughter of pregnant animals in Nigeria, may have also encouraged SPGs [41]. The law is both obsolete and poorly enforced. This law enacted over three decades ago it is not up-to-date or in tandem with the current practices and realities in the livestock production and processing sub-sectors in Nigeria, hence gravid animals are usually slaughtered with little or no caution and the culprits are not usually reprimanded.

The 35.5% PGs slaughtered is higher than 22.6% [42] and 27.4% [21] slaughtered in Plateau and Taraba States, Nigeria, respectively; but lower than 62.8% [22] reported in Abia State, Nigeria. At the international level, the 35.5% is lower than 49.7% [43] and 40.2% [44] reported in Ghana and Tanzania, respectively; but higher than 32.8% [45] found in Ethiopia. The slight discrepancies or vast difference in the proportions of PGs slaughtered across these cities and countries may be due to variations in the depth of ante-mortem inspection, implementation of animal welfare laws, farmers’ coping strategies against feed/water scarcity and cultural/religious/personal sentiments against maternal slaughter.

Apart from goats, SPFAs and the resultant foetal wastages have been reported in other species across Nigeria as follows: pigs, 9% [46], camels, 22.6% [47], cattle, 16.7% [23] and sheep, 24.7% [21]. This is worrisome considering that the country’s average livestock annual growth rate of 1.85% [42] is too slow to satisfy the animal protein requirements of 217 million Nigerians; with an annual population growth rate of 2.6% [11]. Consequently, some Nigerians’ average daily protein intake may be lower than the recommended 1gram per kilogram body weight per person [48]. Low intake of good quality and wholesome animal protein may result in protein malnutrition; especially among children and adolescents. Protein malnutrition is a major public health problem because it lowers innate immunity and hence the ability to fight infectious diseases—malaria, HIV/AIDS, tuberculosis–which are endemic in Africa [49]. Therefore, strategies to mitigate SPFAs, especially SPGs need to be prioritised in Nigeria to avert the untoward economic and public health implications.

Given that the country is yet to achieve self-sufficiency in the production of animal protein, it is worrisome to imagine the colossal loss of 19,136 kilogram of chevon, valued at of ₦47,841, 000 ($115,838) or ₦34.44 million ($83,700) to caprine foetal wastages in just one of the 37 major cities in Nigeria. One bold step to reverse these avoidable losses is to employ more Veterinarians for proper ante-mortem inspection, including pregnancy diagnosis, for all female animals intended for slaughter. Besides, large amounts of feed stuff (silage or hay) could be stocked during the wet/rainy seasons, when lush pastures are abundant, and then sold at subsidized price to goat farmers during drought; to reduce the sale or SPGs as a result of feed scarcity. Additionally, an amendment of the Nigerian Meat Edict of 1988 in line with the current realities in the slaughterhouses and strict implementation of the law thereafter are imperative.

Beside the economic losses, the SPGs may aid transmission of zoonotic pathogens inhabiting the female reproductive tract of the slaughtered animals. *Brucella* (particularly *Brucella melitensis*), *Toxoplasma* and *Leptospira* species are zoonotic pathogens transmissible during processing of infected goat carcasses [14–18, 50]. Apart from these pathogens, Q-fever, Orf and Rift valley fever viruses can spread from infected goats to humans during handling/slaughter [51–53]. In Nigeria, the chances of contamination of the processed meat and or infection of slaughterhouse workers with these zoonotic pathogens are high; considering the unsanitary conditions of the slaughter facilities and non-use of personal protective equipment by the workers during abattoir operations [50]. Moreover, the abattoir environment may also be contaminated with the pathogens. Food-producing animals grazing within the contaminated slaughterhouse environment may then become infected and further transmit the zoonotic organisms to humans, via the food chain.

Apart from the transmission of zoonoses, the ethical and animal welfare issues surrounding SPGs need to be considered. Although the welfare need of foetuses in utero is an emerging aspect in animal welfare science, the possible infringement on the welfare of foetuses as a result of maternal slaughter cannot be completely ruled out. Intense scientific debates have been on-going on whether foetuses perceive pain during maternal slaughter and if, at what gestational age do the pain perception starts. Animals/foetuses need both sentience and consciousness to be able to perceive pain. All through the gestational periods, foetuses are kept in a sleep-like or comatose state via the activities of neuroinhibitors [54]. Reports that consciousness and responsiveness only begin postnatal [55] suggest that during the unforeseen death of the dam, the foetus simply passes from the sleep-like mode to death with minute or no distress at all. However, recent scientific evidences from developmental neuroscience indicate that pain perception in foetuses may commence during the latter stage of the first trimester or at the second trimester; as the brain cortex and the associated tracts, regulating pain perception and responsiveness, usually develop and become functional at mid-gestation [55–58]. In view of the emerging evidence that pain, distress or other forms of suffering may be inflicted on foetuses during maternal slaughter, especially at the third trimester, it may be apt to rethink the SPGs and indeed SPFAs generally because of the animal welfare issues and economic wastages thereof. Thus, new legislation against maternal slaughter to preserve livestock resources and protect the welfare of the foetuses may be worthwhile.

This is important because the SPGs for meat is unprofitable in many ways. The goat farmers, who may have mistakenly culled their animals for infertility reasons due to poor proficiency in pregnancy detection, are at loss. The butchers and goat meat sellers are also at loss because PGs, particularly those in their late stage of pregnancy, yield less meat than non-pregnant ones [59]. Furthermore, the quality of meats sourced from gravid females is doubtful as it may have poor eye appeal, smells and tastes abnormal, due to high progesterone tissue level [1].

The seasonal disposition observed in the SPGs presents useful epidemiological clues that could be exploited to effectively control the practice. For instance, large amounts of animal feed could be stock pilled and sold at low prices to goat farmers during the dry season, to limit feed-scarcity-induced sale or slaughter of PGs. Significant loss of body condition, due to drought-related feed and water scarcities during the dry/hot season; usually compels goat farmers to salvage their animals by selling them off, not minding their pregnancy statuses [42]. Moreover, increased production of foods of animal origin, especially poultry that has a short generation interval, could boost meat supply and reduce meat scarcity/demand that prompt farmers to sell off their goats, including pregnant ones, as buyers tend to offer higher prices during the festive periods.

## Conclusion

About one in every three (35.5%) female goats slaughtered in Enugu, Nigeria was pregnant leading to the loss of 907 foetuses within the six months this study duration. About ₦34.44 million ($83,390) would have been earned if the foetuses were born alive and raised to maturity. Additionally, 19,136 kg of chevon, valued at ₦47,841, 000 ($115,838), which would have accrued from the wasted foetuses was also lost due to the indiscriminate SPGs. Economic hardship, ignorance of the pregnancy status of the slaughtered does, increase demand for chevon and draught-induced feed scarcity were the major reasons for the slaughter. In view of the number of PGs slaughtered and the foetuses wasted; there is need for an amendment and stringent implementation of the Meat Edict of 1988 to limit the negative consequences of indiscriminate slaughter of gravid animals. The amendment may include making pregnancy diagnosis compulsory in pre-mortem inspection protocol for all female goats intended for slaughter in the study area. Furthermore, labelling of meats sourced from PGs is recommended to guide the meat buyers’ discretion. Slaughterhouse workers flouting the laws/regulations could be publicly reprimanded to deter intending violators. In addition, large amounts of feed stuff could be stocked and sold at subsidized rates to goat farmers during the dry season, as an incentive to reduce the sale or SPGs as a result of feed scarcity. Furthermore, public enlightenment campaigns against SPGs and training of livestock farmers and slaughterhouse workers on some on-farm and rapid pregnancy diagnostic methods could help to further ameliorate the situation in the study area. These could reduce the number of PGs slaughtered, minimise the consequential economic and foetal wastages, grow the national goat herd-size, improve food safety/security, and limit the dissemination of zoonotic pathogens in the study area.

## Limitations of the study

The dentition method used for the determination of the ages of the goats slaughtered may not be error free and therefore may have provided only an estimation of the actual ages. Additionally, economic loss estimation was based on the market price of matured goats. Although 5% pre-maturity mortalities and cost of the goat production to market size were factored-in during the estimation, inaccuracies leading to overestimation or under-estimation of the losses may be possible. The authors couldn’t verify the age claims of the respondents but it’s unlikely that minors may work as butchers in slaughterhouses due to the tedious nature of the job. Therefore, results on the age of the dams and financial losses should be interpreted with caution.

## Supporting information

S1 Table Summary of raw data obtained during the survey for slaughter of pregnant goats for meat in Enugu, Nigeria. (DOC)

S1 File (DOC)

S2 File (DOC)
