# Supplementary material for: Indiscriminate slaughter of pregnant goats for meat in Enugu, Nigeria: Causes, prevalence, implications and ways-out
Source: PLoS One. 2023 Jan 17;18(1):e0280524. doi: 10.1371/journal.pone.0280524 (PMC9844864; doi:10.1371/journal.pone.0280524)
Supplement: S1 Table — (DOC) [file pone.0280524.s001.doc]

**Table S1: Summary of raw data obtained during the survey for slaughter of pregnant goats for meat in Enugu, Nigeria**

Overview of the number, pregnancy status and number of foetuses recovered from does slaughtered for meat in Enugu, Nigeria

| **Slaughterhouse** | **Number of does slaughter** | **Number selected and surveyed** | **Number pregnant** |
| --- | --- | --- | --- |
| Akwata | 1,145 | 789 | 271 |
| Artisan | 1415 | 869 | 318 |
| Total | 2560 | 1658 | 589 |

Pregnancy types, Foetal gestational age and sexes found in does slaughtered for meat in Enugu, Nigeria

| **Type of pregnancy** | | | **Gestational age of foetuses (trimester)** | | | **Foetal sex** | |
| --- | --- | --- | --- | --- | --- | --- | --- |
| Singleton | Twin | Triplet | First (< 50 days) | Second (50-100 days) | Third (> 100 days) | Male | female |
| 312 | 236 | 41 | 332 | 486 | 89 | 399 | 508 |

Seasonal distribution of pregnant goats slaughtered for meat in Enugu, Nigeria

| **Season** | **Number surveyed** | **Number pregnant** |
| --- | --- | --- |
| Dry/hot season (January to March ) | 1007 | 318 |
| Wet/ rainy season ( June to July) | 651 | 271 |
| Total | 1658 | 589 |

Age distribution of pregnant goats slaughtered for meat in Enugu, Nigeria

| **Age** | **Number surveyed** | **Number pregnant** |
| --- | --- | --- |
| ≤ 4 years | 876 | 288 |
| > 4 years | 782 | 301 |
| Total | 1658 | 589 |

Breed distribution of pregnant goats slaughtered for meat in Enugu, Nigeria

| **Breed** | **Number surveyed** | **Number slaughtered** |
| --- | --- | --- |
| Sahel | 181 | 83 |
| Red Sokoto | 727 | 241 |
| Kano brown | 618 | 216 |
| West African dwarf goat | 132 | 49 |
| Total | 1658 | 589 |
