# Supplementary material for: Indiscriminate slaughter of pregnant goats for meat in Enugu, Nigeria: Causes, prevalence, implications and ways-out
Source: PLoS One. 2023 Jan 17;18(1):e0280524. doi: 10.1371/journal.pone.0280524 (PMC9844864; doi:10.1371/journal.pone.0280524)
Supplement: S1 File — (DOC) [file pone.0280524.s002.doc]

**UNIVERSITY OF NIGERIA, NSUKKA**

**Faculty of Veterinary Medicine**

**Questionnaire survey on reasons for sale or slaughter of pregnant goats for meat and methods of disposal of the eviscerated foetuses among goat carcass processors and goat farmers/sellers in Enugu State, Nigeria**

Please provide your responses by ticking the most appropriate option in the response column

| **S/n** | **Questions/information required** | **Responses** |
| --- | --- | --- |
| **SOCIO-ECONOMIC CHARACTERISTICS OF RESPONDENTS** | |  |
| **1.** | **Gender** |  |
|  | Male |  |
|  | Female |  |
| **2.** | **Age Category** |  |
|  | Less than 45 years |  |
|  | ≥ 45 years |  |
| **3.** | **Marital status** |  |
|  | Single |  |
|  | Married |  |
| **4.** | **Job description** |  |
|  | Goat carcass processor/Goat meat seller |  |
|  | Goat seller/farmer |  |
| **6.** | **Work experience** |  |
|  | Less than 10 years |  |
|  | ≥ 10 years |  |
| **7.** | **Highest educational level attained** |  |
|  | Tertiary education |  |
|  | Primary and Secondary education |  |
|  | No formal education |  |
| **8.** | **Have had formal training on modern goat farming or hygienic carcass processing** |  |
|  | Yes |  |
|  | No |  |
| **REASONS FOR SALE OR SLAUGHTER OF PREGNANT GOATS FOR MEAT** | | |
| **9.** | **Have ever sold or slaughtered pregnant goat for meat** |  |
|  | Yes |  |
|  | No |  |
| **10.** | **If yes, what was your major reason for doing that?** |  |
|  | Ignorance of the pregnancy status |  |
|  | Economic hardship |  |
|  | Buyers preference for large-sized goats |  |
|  | Feed scarcity due to prolonged drought |  |
|  | Emergency slaughter due to injuries and diseases |  |
|  | Increased demand in goat meat during festive periods |  |
|  | Others (please specify) |  |
| **DISPOSAL OF EVISCERATED FOETUS OR THE GRAVID** | |  |
| **11.** | **Have ever eviscerated caprine foetuses during carcass dressing** | |
|  | Yes |  |
|  | No |  |
| **12.** | **If yes, what was your major means of disposing the eviscerated foetus or the gravid uterine contents?** | |
|  | Sold for preparation of dog food |  |
|  | Sold for human consumption |  |
|  | Discarded by open refuse dump in the slaughterhouse environment/ municipal waste bin | |
|  | Fed raw to pigs and cultured fishes |  |
|  | No response |  |
