# Supplementary material for: Indiscriminate slaughter of pregnant goats for meat in Enugu, Nigeria: Causes, prevalence, implications and ways-out
Source: PLoS One. 2023 Jan 17;18(1):e0280524. doi: 10.1371/journal.pone.0280524 (PMC9844864; doi:10.1371/journal.pone.0280524)
Supplement: S2 File — (DOC) [file pone.0280524.s003.doc]

CALCULATING CONTENT VALIDITY INDEX (CVI) for Relevance (E) and Clarity (e)

| Item | E-1 | E-2 | E-3 | E-4 | E-5 | E-6 | e-1 | e-2 | e-3 | e-4 | e-5 | e-6 | (E) 3 or 4  for all | (e) 3 or 4  for all | (E)NOA | (e)NOA | (E)I-CVI | (e)I-CVI |
| --- | --- | --- | --- | --- | --- | --- | --- | --- | --- | --- | --- | --- | --- | --- | --- | --- | --- | --- |
| 1 | 4 | 4 | 4 | 4 | 4 | 4 | 4 | 4 | 4 | 4 | 4 | 4 | YES | YES | 6 | 6 | 1.00 | 1.00 |
| 2 | 4 | 4 | 4 | 4 | 4 | 4 | 4 | 4 | 4 | 4 | 4 | 4 | YES | YES | 6 | 6 | 1.00 | 1.00 |
| 3 | 4 | 4 | 4 | 4 | 4 | 4 | 4 | 4 | 4 | 4 | 4 | 4 | YES | YES | 6 | 6 | 1.00 | 1.00 |
| 4 | 4 | 4 | 4 | 4 | 4 | 4 | 4 | 4 | 4 | 4 | 4 | 4 | YES | YES | 6 | 6 | 1.00 | 1.00 |
| 5 | 4 | 4 | 4 | 4 | 4 | 4 | 4 | 4 | 4 | 4 | 4 | 4 | YES | YES | 6 | 6 | 1.00 | 1.00 |
| 6 | 4 | 4 | 4 | 4 | 4 | 4 | 4 | 4 | 4 | 4 | 4 | 4 | YES | YES | 6 | 6 | 1.00 | 1.00 |
| 7 | 4 | 4 | 4 | 4 | 4 | 4 | 4 | 4 | 4 | 4 | 4 | 4 | YES | YES | 6 | 6 | 1.00 | 1.00 |
| 8 | 4 | 4 | 4 | 4 | 4 | 4 | 4 | 4 | 4 | 4 | 4 | 4 | YES | YES | 6 | 6 | 1.00 | 1.00 |
| 9 | 4 | 4 | 4 | 4 | 4 | 4 | 4 | 4 | 4 | 4 | 4 | 4 | YES | YES | 6 | 6 | 1.00 | 1.00 |
| 10 | 4 | 4 | 4 | 4 | 4 | 4 | 4 | 4 | 4 | 4 | 4 | 4 | YES | YES | 6 | 6 | 1.00 | 1.00 |
| 11 | 4 | 4 | 3 | 4 | 4 | 4 | 4 | 4 | 4 | 4 | 4 | 4 | YES | YES | 5 | 6 | 0.83 | 1.00 |
| 12 | 4 | 4 | 3 | 4 | 4 | 4 | 4 | 4 | 4 | 4 | 4 | 4 | YES | YES | 5 | 6 | 0.83 | 1.00 |
|  |  |  |  |  |  |  |  |  |  |  |  |  | 12/12  S-CVI = 1.0 | 12/12  S-CVI = 1.0 |  |  | Mean i-CVI = 0.97 | Mean i-CVI = 1.00 |

**Relevant and Clarity**: Mean i-CVI or S-CVI should be ≥ 0.9 for the scale or item index to be good enough. If both are, then, it’s excellent. If either Mean i-CVI or S-CVI is < 0.9, then effect the recommended corrections in the questionnaire to enhance clarity or relevance or both, as the case may be.
